# A Self-Applied Multi-Component Psychological Online Intervention Based on UX, for the Prevention of Complicated Grief Disorder in the Mexican Population During the COVID-19 Outbreak: Protocol of a Randomized Clinical Trial

**DOI:** 10.3389/fpsyg.2021.644782

**Published:** 2021-03-29

**Authors:** Alejandro Dominguez-Rodriguez, Sofia Cristina Martínez-Luna, María Jesús Hernández Jiménez, Anabel De La Rosa-Gómez, Paulina Arenas-Landgrave, Esteban Eugenio Esquivel Santoveña, Carlos Arzola-Sánchez, Joabián Alvarez Silva, Arantza Mariel Solis Nicolas, Ana Marisa Colmenero Guadián, Flor Rocio Ramírez-Martínez, Rosa Olimpia Castellanos Vargas

**Affiliations:** ^1^Health Sciences Area, Valencian International University, Valencia, Spain; ^2^Facultad de Psicología, Universidad Nacional Autónoma de México, Mexico City, Mexico; ^3^Iztacala College of Higher Education, National Autonomous University of Mexico, Mexico City, Mexico; ^4^Department of Social Sciences, Universidad Autónoma de Ciudad Juárez, Ciudad Juárez, Mexico; ^5^Independent Researcher, Ciudad Juárez, Mexico; ^6^Independent Researcher, Tijuana, Mexico

**Keywords:** grief, COVID-19, online intervention, user experience, randomized controlled (clinical) trial

## Abstract

**Background:** COVID-19 has taken many lives worldwide and due to this, millions of persons are in grief. When the grief process lasts longer than 6 months, the person is in risk of developing Complicated Grief Disorder (CGD). The CGD is related to serious health consequences. To reduce the probability of developing CGD a preventive intervention could be applied. In developing countries like Mexico, the psychological services are scarce, self-applied interventions could provide support to solve this problem and reduce the health impact even after the pandemic has already finished.

**Aims:** To design and implement a self-applied intervention composed of 12 modules focused on the decrease of the risk of developing CGD, and increasing the life quality, and as a secondary objective to reduce the symptomatology of anxiety, depression, and increase of sleep quality. The Intervention Duelo COVID (Grief COVID) follows the principles of User Experience (UX) and is designed according to the needs and desires of a sample of the objective participants, to increase the adherence to the self-applied intervention, considered one of the main weaknesses of online interventions.

**Methods:** A Randomized Controlled Trial will be conducted from the 22nd of December of 2020 to the first of June 2021. The participants will be assigned to an intervention with elements of Cognitive Behavioral Therapy, Acceptance and Commitment Therapy, Mindfulness and Positive Psychology. The control group will be a wait-list condition, that will receive the intervention 1.5–2 months after the pre-measurement were taken. The Power Size Calculation conducted through G^*^Power indicated the need for a total of 42 participants, which will be divided by 21 participants in each group. The platform will be delivered through responsive design assuring with this that the intervention will adapt to the screen size of cellphones, tablets, and computers.

**Ethics and Dissemination:** The study counts with the approval of the Research Ethics Committee of the Autonomous University of Ciudad Juárez, México, and it is registered in Clinical Trials (NCT04638842). The article is sent and registered in clinical trials before the recruitment started. The results will be reported in future conferences, scientific publications, and media.

## Introduction

### Background

The Coronavirus disease 2019 (COVID-19) pandemic continues indefinitely, and the number of cases is growing exponentially worldwide. Even though more about this virus is being learned every day, there are still several questions about how the disease behaves and why one of its consequences is the high mortality that it causes, mainly in people over 60 years old (Serra Valdes, 2020). It has been proven that advanced age, the presence of diabetes mellitus, hypertension and obesity significantly increases the risk of hospitalization and death in patients with COVID-19 (Muniyappa and Gubbi, 2020).

According to the World Health Organization (WHO), the number of infected people and deaths caused by this virus as of December 19, 2020 has risen to 74,299,042 and 1,669,982, respectively (World Health Organization, 2020).

The pandemic has caused psychological consequences (Li et al., 2020), among which fear, anxiety, post-traumatic stress disorder, depression, suicidal or addictive behaviors stand out, as well as domestic violence as a collateral effect of the confinement (Mengin et al., 2020). Also, financial difficulties could reduce the access to receive mental health treatment (Sher, 2020).

In addition, among the psychological consequences, the grief due to the loss of loved ones cannot be ignored, and it should also be noted the importance of the suffering derived from the mandatory physical separation of family members who are sick with COVID-19 (Singer et al., 2020).

The grieving process has been studied and analyzed by various authors, the psychiatrist Kübler-Ross stands out among the experts on this topic, who defined the five phases in which a person transits during grief: denial, anger, pact/negotiation, depression, and acceptance (Miaja Ávila and Moral De La Rubia, 2013). A person can go through these phases in different ways, therefore, there is no correct way to grieve, since each experience is unique, individual, and requires necessary and adequate support in each particular situation.

Grief is a common human response to loss (Archer, 2003; Weir, 2020). Most people adapt to the death of their loved ones and the changes that occur in their lives (Goveas and Shear, 2020). At the present time, grief is being experienced in different ways, on the one hand there is some uncertainty in the face of daily losses, such as social distancing, economic losses, health losses and the lack of contact with family and friends. On the other hand, there is anticipatory grief, usually is the normal grief that appears when the death of a relative or of oneself is feared (Shore et al., 2016), and it can be experienced in the form of high concern for other people who may be affected due to the disease (Wallace et al., 2020).

As the pandemic progresses and with it the large number of deaths, as well as the lack of preparation for the imminent death of close relatives, the recommended restrictions to reduce the infection and transmission of the virus, the physical, mental, and social consequences of distancing, such as not being able to say goodbye to loved ones, and therefore to not celebrate the traditional social and cultural rituals of grief (Goveas and Shear, 2020; Morris et al., 2020), sometimes necessary to heal the wound caused by the death of the loved person, the pathological or chronic grief may appear complicated (Lobb et al., 2010; Wallace et al., 2020).

Complicated grief is characterized by intense emotional distress that can last longer than socially expected and that causes a disability in the person's daily functioning, endangers their health and well-being, and can last for years and even become indefinitely chronic (Barreto et al., 2012). There are other factors that can contribute to increase it, such as sudden or traumatic death, which results in the lack of preparation for it and the lack of social support at the time of the event (Burke and Neimeyer, 2014). These are factors present at this moment with COVID-19 and they may have a significant impact on the individual and on the social experience of death and grief due to the measures of social isolation and the lack of the usual support structures (Mayland et al., 2020).

Recognizing the uniqueness of each individual with respect to their process of loss and pain will provide opportunities to develop personalized strategies that facilitate psychological flexibility (Hayes et al., 2015) and functional adaptation to the loss, which will promote mental health and well-being in this crisis (Zhai and Du, 2020).

It is therefore clear that grief is inevitable and multidimensional for people with losses. The loss of a loved one is perhaps one of the most shocking events that occur in a person's life (Zhai and Du, 2020). In few periods of human history, grief and pain have been as present in people's lives as they are today (Goveas and Shear, 2020).

It should be noted, considering the reviewed bibliography, that grief will not always need a psychotherapeutic approach, since most people who experience the loss of a loved one cope with their grief in a natural way and without emotional discomfort implying a deterioration in their daily functioning, managing over time to continue with their lives and their activities (Neimeyer et al., 2002; Neimeyer, 2014).

On the other hand, survivors experience a series of consequences in their health (physical and mental) and social interaction that make it difficult for them to continue with their daily lives and lead to considerable wear and tear that triggers the development of complicated grief (Prigerson, 2004), or a mental disorder, such as depression (Boelen and Prigerson, 2007) or post-traumatic stress disorder (Payás, 2010; Christiansen et al., 2013), and in more severe cases, suicidal behavior (Szanto et al., 2006).

In this sense, Litz et al. (2014), suggest that it is possible to prevent complicated grief by intervening in the early stages for people who present significant preclinical symptoms after the loss, reducing the possibility of developing a considerable deterioration in their loss during the following months, functioning on the daily basis and allowing to alleviate emotional suffering gradually.

Regarding the evidence of effective psychological interventions that could be implemented to provide support to the population that suffered a loss, it is important to start with Cognitive Behavioral Therapy (CBT). The CBT proposes that the way we think affects the way we feel and behave (Litz et al., 2014). Thus, helping people to learn how to evaluate their thinking and generate more realistic or accurate thought patterns improves both their emotional and behavioral state (Beck, 2006). The cognitive model provides a framework to identify and challenge inappropriate thoughts or beliefs that can lead to feelings of guilt, anger, or rage (Bayés, 2006). Yahya and Khawaja (2020) study found that internet-based CBT is effective in a series of small randomized controlled trials.

In addition, CBT can be nourished by the practice of Mindfulness. Jon Kabat-Zinn is recognized for being, mainly, one of the first authors who introduced Mindfulness within the field of Western psychology, developing the Mindfulness-Based Stress Reduction Program (MBSR). Mindfulness has become an allied technique of psychotherapy, more focused on acceptance than on change (Simón, 2010). According to Hayes et al. (2006), the component of acceptance of cognitions and sensations in Mindfulness would decrease emotional reactance and would allow a healthier and more effective coping in patients subjected to some type of trauma, such as grief. In relation to mindfulness-based treatments that have been highly investigated in recent years, a systematic review published by Goldberg et al. (2017), corroborated that Mindfulness-Based Interventions (or MBI) are effective for depression, grief, and pain conditions, smoking, and other addictions. The basis of these programs is not to change the patient's experience, promoting psychological acceptance by giving importance to the values of the patient or the therapist. It allows patients to learn skills, reduce worry, ruminant thoughts, and emotional cognitive reactivity. They are programs that are carried out in a group mode and can improve the quality of life of patients in a broad sense (Segal et al., 2004).

Other effects of bereavement include loss of pleasure and interest in activities (Craske et al., 2019). Focused attention on the meaning of the loss, positive reinforcement for the actions taken (Bartone et al., 2019), behavioral activation techniques, such as activities, reinforcement of self-care and contact and support among the peer group (Lacasta and Aguirre, 2020) and the performance of rituals, attention to spirituality and the need for a farewell are presented as the central axis to avoid complicated grief (Barbero et al., 2014).

It should be noted that although Psychotherapy in Mexico is mainly carried out in private practice, it is expanding to public institutions, like hospitals and ambulatory clinics (Sánchez-Sosa, 2007), however mental health services are still insufficient (Martinez et al., 2017).

Zhang et al. (2006), proposed that clinical interventions can focus on the following elements: (a) differentiate between expected grief reactions and those of complicated grief; (b) detect the risk factors that make people more vulnerable to develop complications in grief; and (c) establish intervention actions to prevent maladaptive responses to loss.

Taking into account the reduction in the availability of carrying out psychological interventions, it has been selected to provide psychological support at a distance through different communication networks and platforms (Eisma et al., 2020). These interventions can be applied if the pandemic continues for longer periods. During the last decades, an increase in the implementation of online interventions has been observed due to the advantages that this entails, such as having greater flexibility and anonymity, in addition to having demonstrated positive effects comparable to face-to-face therapy (Wagner et al., 2014; Hoffmann et al., 2018).

On other hand, self-applied interventions appear as an option to arrive at a great number of participants. To make a self-applied intervention effective, tools are included to communicate skills that help to externalize the problem and set realistic goals, as recommended by Malkinson (2010), with the cross-cutting objective of reducing anxiety symptoms and exploring emotions (Aoun et al., 2020; Morris et al., 2020). Along the same lines, previous research has shown the effectiveness of online tools in grief support that help improve the adaptive adjustment of people in grief (Dominick et al., 2010). There is evidence of the efficacy of online interventions applied to patients suffering from abnormal grief. For example, in studies where an online intervention was carried out in which the patient has to write a letter to the deceased person, the researchers concluded that this activity alone was efficient in reducing emotional loneliness and increasing the state of positive mood, as well as its effect on rumination; however, no effects were observed in terms of grief and depression symptoms (Van der Houwen et al., 2010), indicating that an online intervention aimed at this population should be multicomponent.

In the study by Kersting et al. (2013) a brief 5-week intervention based on CBT, was implemented for parents who lost a child during pregnancy. The contents of the intervention were delivered through a web platform focused on three central axes: (a) self-confrontation, (b) cognitive reappraisal, and (c) social sharing. This study had 228 participants divided into two groups (intervention and on the waiting list). The participants in the intervention group reduced the symptoms of post-traumatic stress, prolonged grief, depression, and anxiety, with statistically significant changes (Kersting et al., 2013).

Another study focused on relatives of patients who died from hematological cancer, in which the participants were similarly divided into two conditions, the intervention group and the control group. The intervention group received the contents of the intervention similar to the Kersting study: (a) self-confrontation (patients describe their experience of loss, with a special emphasis on emotional and cognitive processes), (b) cognitive reassessment (the purpose of this phase is to work on a change of perspective to help participants develop realistic and useful coping strategies), and (c) social sharing (in this phase patients have to write a letter for people affected by the death of a loved one, including themselves, and a letter to the person who passed away). The Internet-based grief therapy is assumed to have at least moderate effects regarding Prolonged Grief and other bereavement-related mental health outcomes (Hoffmann et al., 2018). Also, there is wide evidence of the effectiveness of psychological interventions in terms of maintaining the results on the follow-ups, such as 3 months for a self-help online intervention for suicidal thoughts (Van Spijker et al., 2015) and a Meta-Analysis of Cowpertwait and Clarke (2013), identified that web-based interventions for depression where the effects are maintained for 3–6 months, and even the results have been observed to maintain in a 12 months follow-up in a self-help intervention for parents of children on cancer treatment (Cernvall et al., 2017), to mention a few examples.

Along similar lines, online interventions for patients with abnormal grief are supported by studies that have demonstrated their efficacy. However, one of the main problems that have been observed in terms of online interventions aimed at treating depression and anxiety symptoms is the broad description of the theoretical content, and the poor description of the relevant characteristics of the human-computer interaction design (Søgaard and Wilson, 2019). For example, a study published in 2017, identified that there is a lack of research offering qualitative data about the subjective User Experience (UX) of young people using interventions for depression, such as social network based (Santesteban-Echarri et al., 2017). In this sense, in the intervention of this research we have to take this into account and assume that in some cases an impediment may be encountered, such as the user's lack of experience in the use of ICTs or the need for more human contact. It is also important to note that the duration of the entire intervention without time spaces in which to receive feedback on improvement or emotional stability may lead to abandoning of the therapy, or lack of adherence to treatment. It is hoped that results can be seen throughout each module, but there may always be someone with an urgent need for instant feedback on improvement.

Likewise, there is available evidence that online interventions have good adherence to treatment in weekly sessions of 50 min, and with work between sessions. However, this structure is not recommended for groups that are not clinical, for example, for prevention and/or for people with mild problems, since the time they dedicate to the interventions is reduced, or if they do it only once, or if it is unlikely that they will return and finish (Cavanagh, 2010). Also when it comes to interventions of longer periods, there is the risk of losing motivation to continue (Melville et al., 2010), therefore, it is proposed to use short-time videos and to have at least two sessions per week (every other day) in order to increase adherence and probability of completing the intervention.

Among the aspects of human-computer interaction design, the concept of universal design aims to design interactions with digital tools which are aesthetically pleasing, and at the same time ensures that the tool can be used by all participants, regardless of their age, ability, or status (Søgaard and Wilson, 2019). It is relevant to note that older adults are interested in using technology to take care of their mental health and this form of intervention is feasible and reliable for them (Figueroa and Aguilera, 2020). According to these same authors, these interventions are specifically aimed at this vulnerable group and are adapted to their specific needs. It includes easy-to-use design options and uses a vocabulary adapted to the general population. In addition, its infrastructure ensures confidentiality, without violation of privacy and minimizes the risk of data leaks.

Other online interventions are known to mitigate the impact of COVID-19 on health, in which psychological well-being is promoted in health professionals (Blake et al., 2020). It is particularly vital to stimulate the development and dissemination of Internet-based treatments for grief, and it is also a question to ask if they should implement this type of intervention in health care systems. Due to the current circumstances, it is relevant to provide an online intervention to aid the population suffering of Grief due to the loss of a loved one, due to COVID or during the most part of this year, where the measures are strict, and funerals are unrecommended.

The intervention protocol through the platform presented in this research is focused to contribute to the reduction of the development of Complicated Grief Disorder (CGD) after experiencing the traumatic situation of loss, in this case specifically from the contingency of COVID-19 with a self-applied intervention based on CBT, Mindfulness, Behavioral Activation Therapy (BAT), and Positive Psychology (PP) and an increase in the quality of life. Other aims are the reduction of anxiety/depression symptoms and the increase of sleep quality. In this way the survivors will be able to establish self-care measures in different areas of their life (physical, emotional, cognitive, and spiritual), and the risk of the appearance of complicated grief is diminished (Greenberg et al., 2008).

### Aims

The online Intervention Grief COVID (Duelo COVID), aims to provide a self-applied intervention composed of 12 sessions based on CBT, Mindfulness, BAT, and PP, aimed at the decrease of the risk of developing Complicated Grief Disorder (CGD) specifically from the contingency of COVID-19, and increasing the quality of life. And as a secondary objective, to reduce the symptomatology of anxiety, depression, and to increase sleep quality. With the objective that the survivors are be able to establish self-care measures in different areas of their life (physical, emotional, cognitive, and spiritual).

## Hypotheses

### Primary Hypothesis

The self-applied multi-component psychological online intervention for the prevention of complicated grief disorder will show greater improvement in the quality of life and perception of the satisfaction of life than a waitlist control group.

### Secondary Hypothesis

Participants in the self-applied multi-component psychological online intervention will report better indicators of change in reduction of symptoms of anxiety, depression, and greater sleep quality compared to the waiting list group; and the changes will be maintained for 3 and 6 months after completing the treatment.

## Methods and Analysis

### Study Design

**Figure 1:**
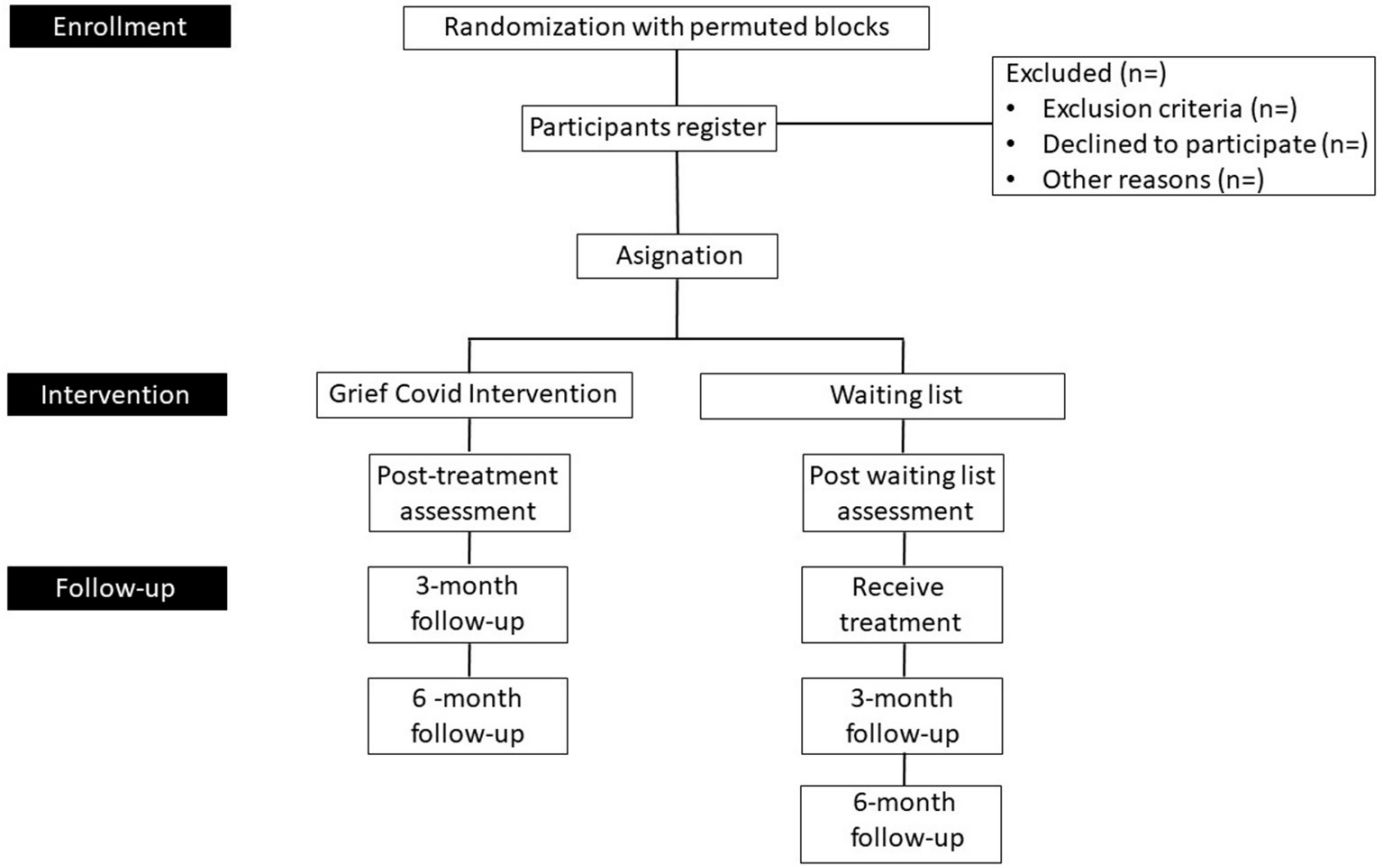
Flow chart of the study design of the “Grief COVID” (Duelo COVID) intervention.

A randomized controlled clinical superiority trial with two independent groups will be used, with intrasubject measures at four evaluation periods: pretest, post-test, follow-up at 3 months, and follow-up at 6 months (Solomon et al., 2009). Participants will be randomly assigned to one of two groups:

1. Grief COVID-19 intervention, participants in this group will receive 12 sessions of a multi-component psychological intervention focused on the decrease of the risk of developing CGD, increasing the life quality, reduction of symptoms of anxiety/depression and increase of sleep quality. Each session will be administered every third day to give time to do the tasks, and not too long to reduce the chance of abandoning the treatment.
2. Waiting List group, the participants in this group will not receive the treatment immediately. They will be measured one time and then a second time 1.5–2 months later than the intervention group when it is calculated that the first group has carried out 12 sessions. The post-measures and follow up will be applied to all the participants to analyze the effectiveness of the intervention (see Figure 1).

### Randomization Process

**Figure 2:**
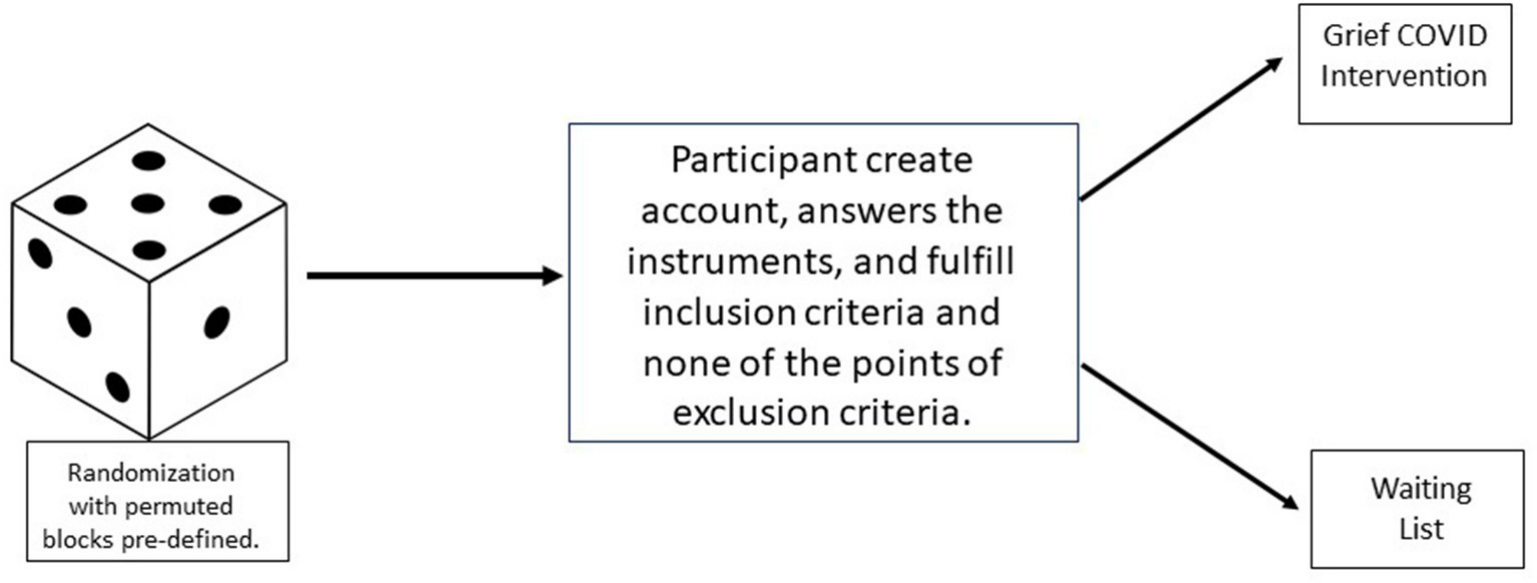
Randomization process of the “Grief COVID” (Duelo COVID) intervention.

This will be a randomized controlled efficacy trial comparing an intervention for grief within control. The randomization procedure will use a permuted blocks algorithm via the Study Randomizer software (Study Randomizer, 2020), where a researcher in the team will obtain the location for the participants before they join the intervention. The process will consist on that once the participant creates an account on the platform, and fulfills the inclusion criteria, and does not fulfill any point of the exclusion criteria, he/she will be assigned to the corresponding condition (see Figure 2).

### Sample

A total of 49 Spanish-speaking male and/or female users meeting the inclusion criteria are expected to be recruited via the online Grief COVID platform. The intervention is aimed at participants aged 18 or older.

### Participant Criteria

Inclusion Criteria:

1. To have a communication device with access to the Internet (computer, tablet, or mobile).
2. To have a valid e-mail address.
3. To have basic digital skills in the use of an operational system and Internet browsing.
4. To be fluent in Spanish, since the complete intervention is in such language.
5. To have symptoms of Depression, State Anxiety and/or Acute Stress Disorder grief symptoms.

Exclusion Criteria:

1. To have a diagnosis of psychotic disorder.
2. To have more than 6 months passed since the death of the loved person.
3. To receive psychological and/or pharmacological treatment during the study.
4. To have a moderate to a high score in the suicide scale.
5. To have a recent attempt of suicide (3 months).
6. To have a diagnosis of Post-traumatic Stress Disorder.

The participants need to meet all the five points of the inclusion criteria to access the intervention and to not meet any of the six points of the exclusion criteria.

### Psychological Measures

#### Center for Epidemiologic Studies Depression Scale (CES-D)

Depression levels will be assessed by the CESD-D, a self-report scale that assesses symptoms of depression in the past 2 weeks. This scale consists of 20 questions and contains four possible answers: rarely or never (<1 day), sometime or rarely (1–2 days), occasionally or a good part of the time (3–4 days) and most of the time (5–7 days). This instrument has been constantly used in health research and its psychometric properties prove to be a valid scale in Mexican population (Cronbach's alpha > 0.90), according to González-Forteza et al. (2011), and among different populations, such as young people and adults (Cuijpers et al., 2007).

#### Depression Anxiety Stress Scale (DASS-21)

The DASS-21 is a self-report scale that assesses the depression, anxiety, and stress subscales during the past week. Each subscale contains seven questions with four possible answers (0–3) as follows: does not apply to me (0), it applies to me to some degree, or sometimes (1), they applied to me to a considerable degree or a good part of the time (2), they applied to me a lot or most of the time (3). All scores must be multiplied by two to obtain the final score, where each subscale has a cut-off score for each severity condition (normal, moderate, and severe), the cut-off point for moderate levels is 14–20 for depression, 10–14 for anxiety, and 19–25 for stress, where any previous score is considered severe or extremely severe (Lovibond and Lovibond, 1995). This scale has been validated in the Mexican population obtaining a reliability for global (α = 0.86) and for each subscale; depression (α = 0.81), anxiety (α = 0.76), and stress (α = 0.79) (Gurrola et al., 2006).

#### The Pittsburgh Sleep Quality Index

The quality of sleep scores will be evaluated using the Pittsburgh Sleep Quality Index. This instrument assesses sleep quality patterns, differentiating people who have poor sleep quality from those who have good sleep quality. For this, seven areas are evaluated, where the response ranges go from 0 to 3 with a total sum that goes from 0 to 60, where the cut-off point is a score of 5, which indicates a poor quality of sleep (Buysse et al., 1989). The evaluation in the Mexican population has shown solid criteria of reliability (α = 0.78) (Jiménez-Genchi et al., 2008).

#### Post-traumatic Stress Disorder Symptom Scale

It is a 17-item structured interview. The severity over the last 2 weeks of each item on the PSS is rated by the interviewer using a 4-point scale: 0 = not at all, 1 = a little bit, 2 = somewhat, and 3 = very much. The maximum possible score is 51 (severely affected) and the minimum possible score is 0 (total absence of the symptoms). The total severity score is calculated as the sum of the severity ratings for the 17 items. The diagnosis is made when one symptom of re-experience, three of avoidance and two of activation are observed (Foa et al., 1997). For this study, the validated version in Spanish will be used (Novy et al., 2001).

#### Satisfaction With Life Scale

This instrument consists of five items in which the participants must indicate how much they agree with each question, with an answer option in Likert format from 1 (totally disagree) to 7 (totally agree), the scores range from a minimum or 5 to a maximum of 35, where the highest scores indicate greater satisfaction with life (Vázquez et al., 2013). This scale has been validated in the Mexican population, obtaining good results of internal consistency (α = 0.74) (López-Ortega et al., 2016).

#### Beck's Hopelessness Scale

This scale is composed of 20 items with a false or true answer option, the score ranges from 0 to 20 with higher scores indicating a higher level of hopelessness (Beck et al., 1974). It is a widely validated and used scale, and for this study the version validated in the Mexican population will be applied (Osnaya and Pérez, 2012).

#### Generalized Anxiety Disorder 7-Item (GAD-7)

The Generalized Anxiety Disorder 7-Item (GAD-7) scale. This is a brief scale consisting of 7 items designed to measure the severity of symptoms of generalized anxiety disorder. The answers are based on the symptoms perceived during the last week. The questions in this scale are answered in a Likert format with scores from 0 to 3, where the maximum total score is 21. A score between 0 and 4 points indicates that anxiety is not perceived, and a score between 15 and 21 is an indicator of perceived severe anxiety (Spitzer et al., 2006). The version in Spanish by García-Campayo et al. (2010), will be used for this study.

#### Inventory of Complicated Grief

It is composed of 19 items, with a five Likert-type scale ranging from 0 to 4, where: 0 “never,” 1 “rarely,” 2 “sometimes,” 3 “often,” and 4 “always.” The items assess the frequency of the explored symptoms type (emotional, cognitive, or behavioral). For its evaluation, the points of each item are added, and the scores fluctuate between 0 and 76 points. Scores above 25 are an indicator of complicated grief. The properties of the adaptation of the scale to Spanish have good results of internal consistency (α = 0.88). The version of Limonero et al. (2009), will be used for this study.

#### World Health Organization Quality of Life (WHOQoL)-BREF Spanish Version

This instrument is composed of 26 items, two global questions (global quality of life and general health), and 24 questions that provide a profile on the responders' life quality in four dimensions: (1) Physical health, (2) Psychological health, (3) Social relationships, and (4) Environment. It focuses on the degree of satisfaction that the person has with various situations in their daily life. Each item has 5 Likert-type response options (1–5). The scale was validated in the Mexican population showing wide validity in clinical settings (Acosta-Quiroz et al., 2013).

#### Plutchik Suicide Risk Scale

This questionnaire assesses the risk of suicide through questions posed in a dichotomous way (yes/no), where the history of suicide attempts, suicidal ideation and suicide are considered plans. This scale establishes a cut-off point of >6 that differentiates people at risk from those who are not at risk of suicide (Plutchik and Van Praag, 1994). The properties of this scale have shown good reliability (α = 0.74), based on these findings, it is established that it is an appropriate questionnaire to assess the risk of suicide. This scale has been used in previous studies with the Mexican population (Alderete-Aguilar et al., 2017).

## Secondary Measures

### Acceptance/Satisfaction/Usability Measures

#### Opinion on the Treatment

This questionnaire is made up of four questions that report the level of satisfaction with the treatment, if the users would recommend the treatment to a friend or relative, if the patient considers the treatment useful, and if they think the treatment was difficult to handle or was aversive. The items are answered on a scale from 1 (not at all) to 10 (a lot) (Botella et al., 2009).

#### System Usability Scale

It is an instrument designed to validate the usability of a system, it is composed of 10 items, which are answered on a 5-point Likert-type scale with respect to the degree of conformity of the product (1 = totally disagree to 5 = completely agree). To obtain the global score of this scale, all the values obtained must be added and multiplied by 2.5, and this will result in a number between 0 and 100, which will be the global value of this scale (Brooke, 1996).

### Study Period

Pre-intervention screening and the intervention process itself shall start on December 22nd and it is expected to conclude by June 1st. By then, the targeted sample is expected to be recruited. Prospective 3- and 6-month follow up assessments on outcome variables will be conducted to ascertain the intervention's effectiveness. The detailed description of the plan of the steps and instruments that will follow this study can be found on Table 1.

**Table 1:** SPIRIT figure to display the study's schedule of enrolment, interventions, and assessments.

	**Study period**					
	**Enrolment**	**Allocation**	**Post-allocation**			
**Timepoint**	**t0**	**0**	**t1: PRE**	**t2: Post**	**t3: Follow-up 1**	**t4: Follow-up 2**
**ENROLMENT**						
Eligibility criteria	X					
Informed consent	X					
Allocation		X				
**INTERVENTIONS**						
1) Grief COVID-19 intervention						
2) Waiting List group						
**ASSESSMENTS**						
**Primary outcome measure**						
Center for Epidemiologic Studies Depression Scale (CES-D)	X			X	X	X
Depression Anxiety Stress Scale (DASS-21)	X			X	X	X
The Pittsburgh Sleep Quality Index	X			X	X	X
Satisfaction with Life Scale	X			X	X	X
Beck's Hopelessness Scale	X			X	X	X
Inventory of Complicated Grief	X			X	X	X
World Health Organization Quality of Life (WHOQoL)-BREF	X			X	X	X
Plutchik Suicide Risk Scale	X					
Post-Traumatic Stress Disorder Symptom Scale	X					
Generalized Anxiety Disorder 7-item (GAD-7)	X					
**Secondary further outcome measures**						
Opinion treatment				X		
System Usability Scale				X		

### Outcomes

- Improved perceived satisfaction with life and quality of life is expected upon completion of the intervention.
- A reduction of anxiety and depression symptoms, as well as an increase of sleep quality are expected upon completion of the intervention. Such changes are expected to be maintained 3 and 6 months after the end of the intervention process.

### Description of the UX Process for the Design of the Grief COVID Platform

This intervention was created following the principles of UX, ensuring that the design characteristics of the tool will meet the desirable requirements to be perceived as easy to use, attractive and useful by the participants. The UX approach refers to the experience that a user has with a product, with special emphasis on human-product interaction (Hassenzahl, 2008; Tullis and Albert, 2013). The UX process was conducted by the main author who is a certified UX designer.

The first step was to review similar interventions, but there was none found that fulfilled the goals of the COVID Grief intervention. Afterwards, six interviews were conducted through Zoom with six objective users (persons that lost someone due to COVID or during the COVID outbreak) through interviews with a duration of 30–40 min where they shared what contents they would like on the platform. The interviews were recorded with the informed signed consent of the participants in order to evaluate with more details the information of the intervention.

After analyzing the recordings of the interviews, affinity mappings were conducted to find similar requests, needs or suggestions from the participants toward the intervention. From the results User Personas were created, these are fictional characters based on the overall results of the participants interviews (Adlin et al., 2006). Afterwards User Journey Maps and User Flows were also created, along with a site map proposal with four main sections: (1) Sessions, (2) Talk with a Psychologist, (3) Technical Support, and (4) My Profile.

Furthermore, a card sorting test was distributed online through Optimal Workshop (2020), and 13 participants completed the task. Also, the site map was redefined to three sections where the options “Talk with a Psychologist” and “Technical Support” were fitted into the “Help center” section. Afterwards, wireframes with the first proposal of the platform were drawn, followed by the creation of a low-fidelity prototype in Balsamiq (2020), followed by the creation of a mid-fidelity prototype in Figma (2020), a collaborative web design tool based in a web browser. The mid-fidelity prototype was validated with five objective participants individually through remote testing through zoom, they were also recorded, consent of the participants was obtained, and the usability of the platform was measured with a subjective scale reported by the scale from 0 not usable at all, to 10, totally usable without complications. The evaluated sections were: (1) create an account, (2) the onboarding process, (3) Section where the sessions of the treatment are and to navigate through the first section, (4) Help Center, and (5) My profile. While analyzing the recordings with the results obtained, another affinity map was conducted: The Observations, Positive Quotes, Negative Quotes, Errors, Suggestions and Metrics. Following the Jakob Nielsen Scale (Nielsen, 1994), where: 0 = I don't agree that this is a usability problem at all, 1 = Cosmetic problem only: need not be fixed unless extra time is available on project, 2 = Minor usability problem: fixing this should be given low priority, 3 = Major usability problem: important to fix, so should be given high priority and 4 = Usability catastrophe: imperative to fix this before the product can be released. All the sections with scores from 4 to 1 were modified in that order of priority.

**Figure 3:**
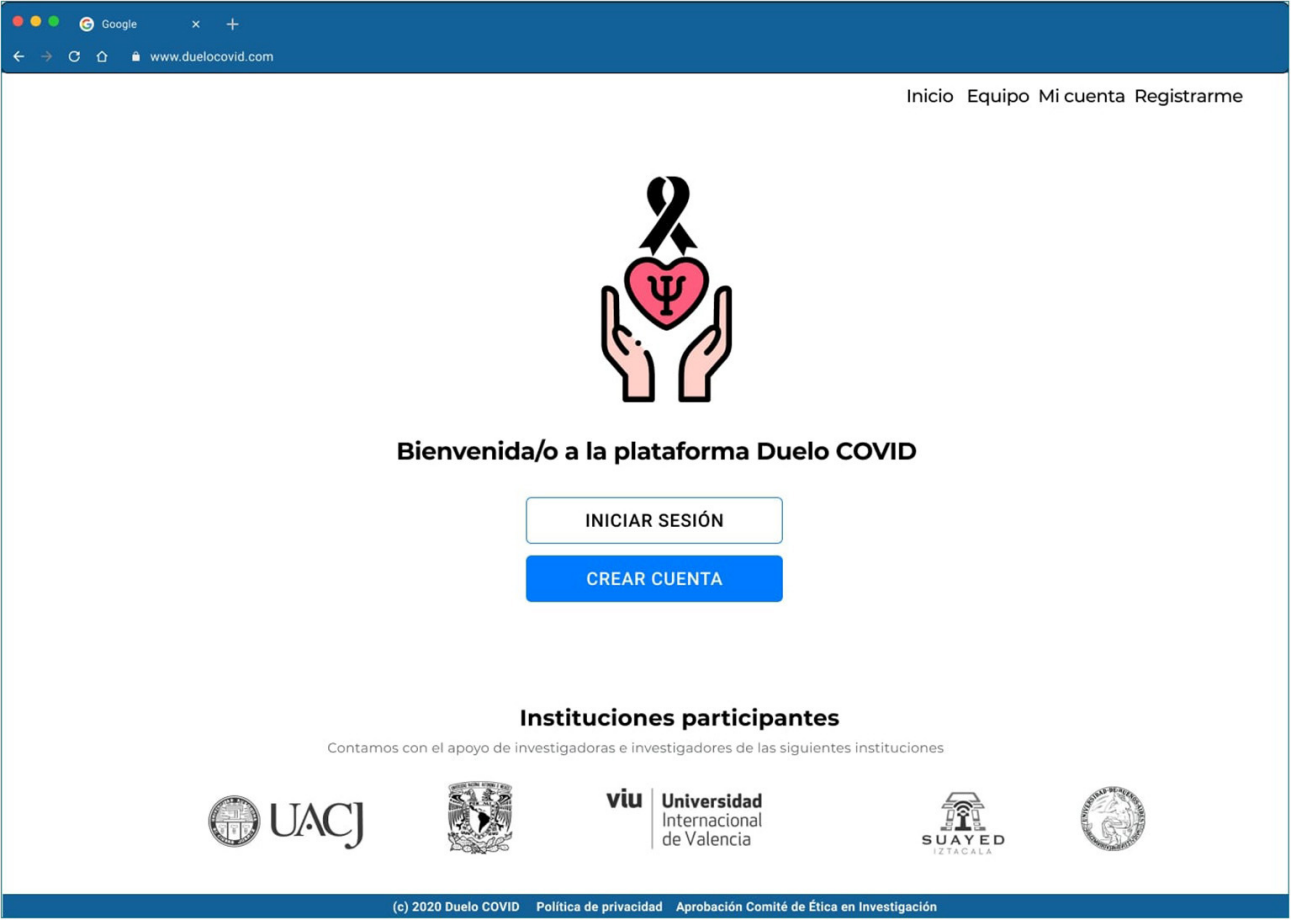
High Fidelity Prototype in Figma of the main page of the “Grief COVID” (Duelo COVID) intervention.

Also, the logo is important to feel connected to the intervention, therefore three options were offered in an online A/B testing distributed thorough Usability Hub (2020), 25 persons participated and 12 chose the logo on Figure 3.

Finally, a high-fidelity clickable prototype was designed in Figma with the previous design inputs by the users. This prototype and their archives were delivered to the Engineer for the development of the platform. The full detail on each step can be found on Domínguez-Rodríguez (2020). The contents of this intervention will be implemented through a responsive web application. The characteristics of this type of system can adapt to different screen sizes and resolutions, from the largest to the smallest screen sizes, such as computers, tablets, and cellphones. This type of tool adapts the page design, resizes images or cuts them proportionally (Baturay and Birtane, 2013). The intervention can be accessed on www.duelocovid.com.

### Structure of the COVID Grief Platform

The platform is designed to be the most usable and simplest possible by every type of user, with or without a wide ability and experience with web platforms or cell phone applications (see Figure 3).

**Figure 4:**
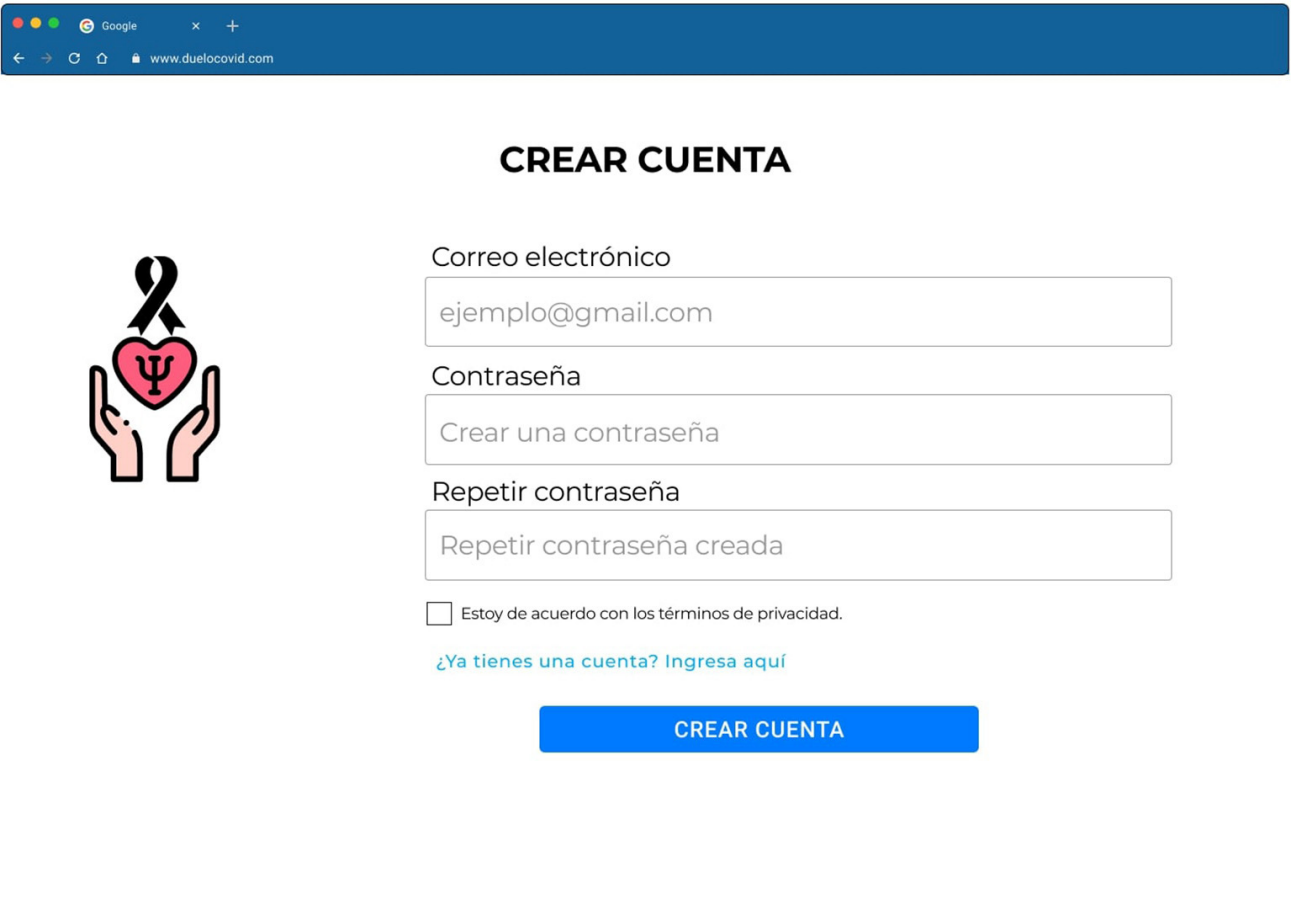
High Fidelity Prototype in Figma of the login/create an account page of the “Grief COVID” (Duelo COVID) intervention.

To create an account on the web page, the participant needs to read and accept the informed consent. Afterwards, the platform just requests an email and password. In order to protect the most possible information of the participant, non-sensible data is requested, such as their name (see Figure 4).

**Figure 5:**
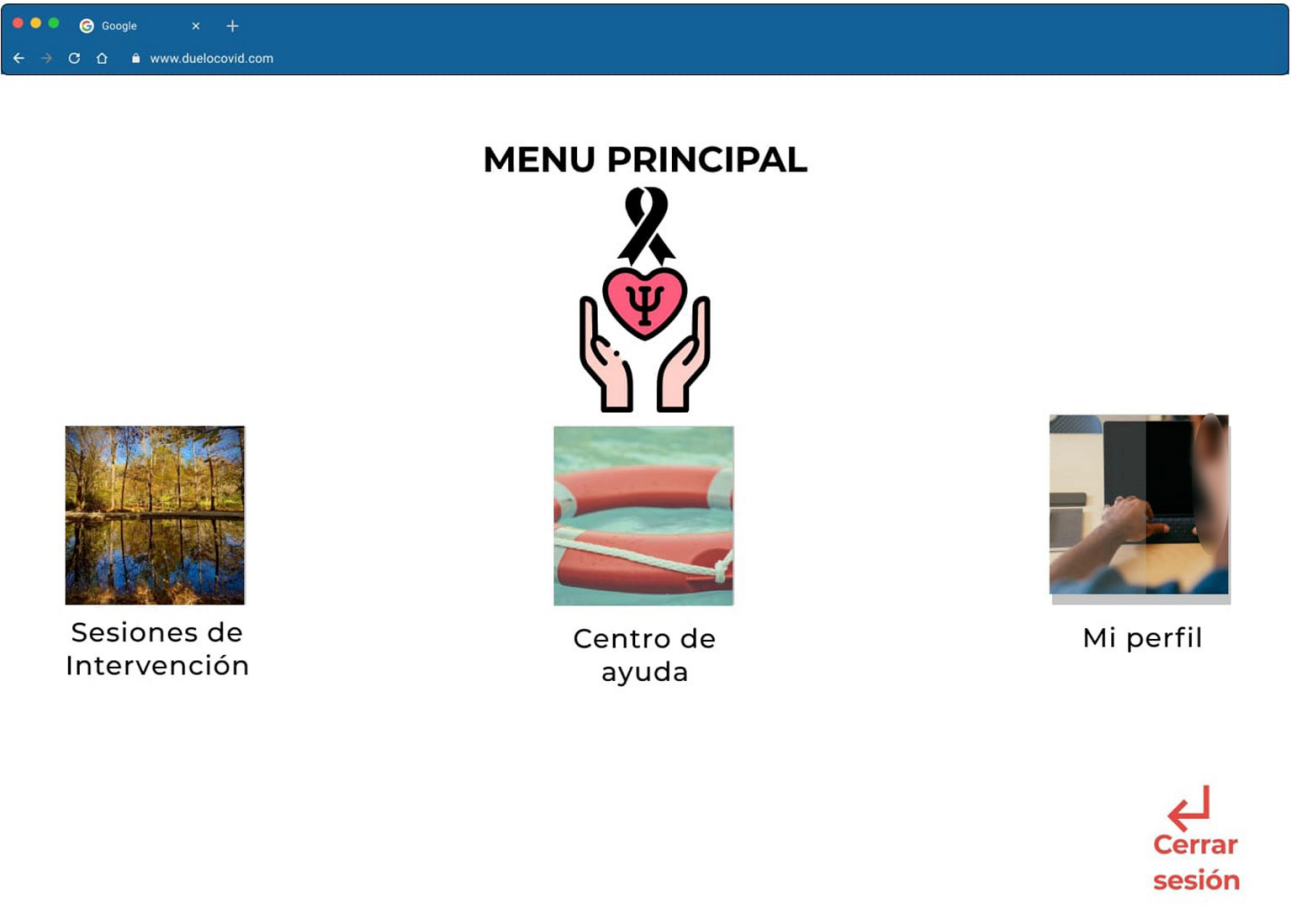
High Fidelity Prototype in Figma of the main menu page of the “Grief COVID” (Duelo COVID) intervention.

Once the participant has created an account, he/she will need to answer the psychometrics, and once finished, he/she can start to use the platform that will begin with an onboarding process, where the platform will explain how it is composed and the sections that are included. Once the onboarding process is finished the participant can find the main menu with the options from left to right: (1) Interventions sessions, (2) Help center, and (3) My profile (Figure 5).

**Figure 6:**
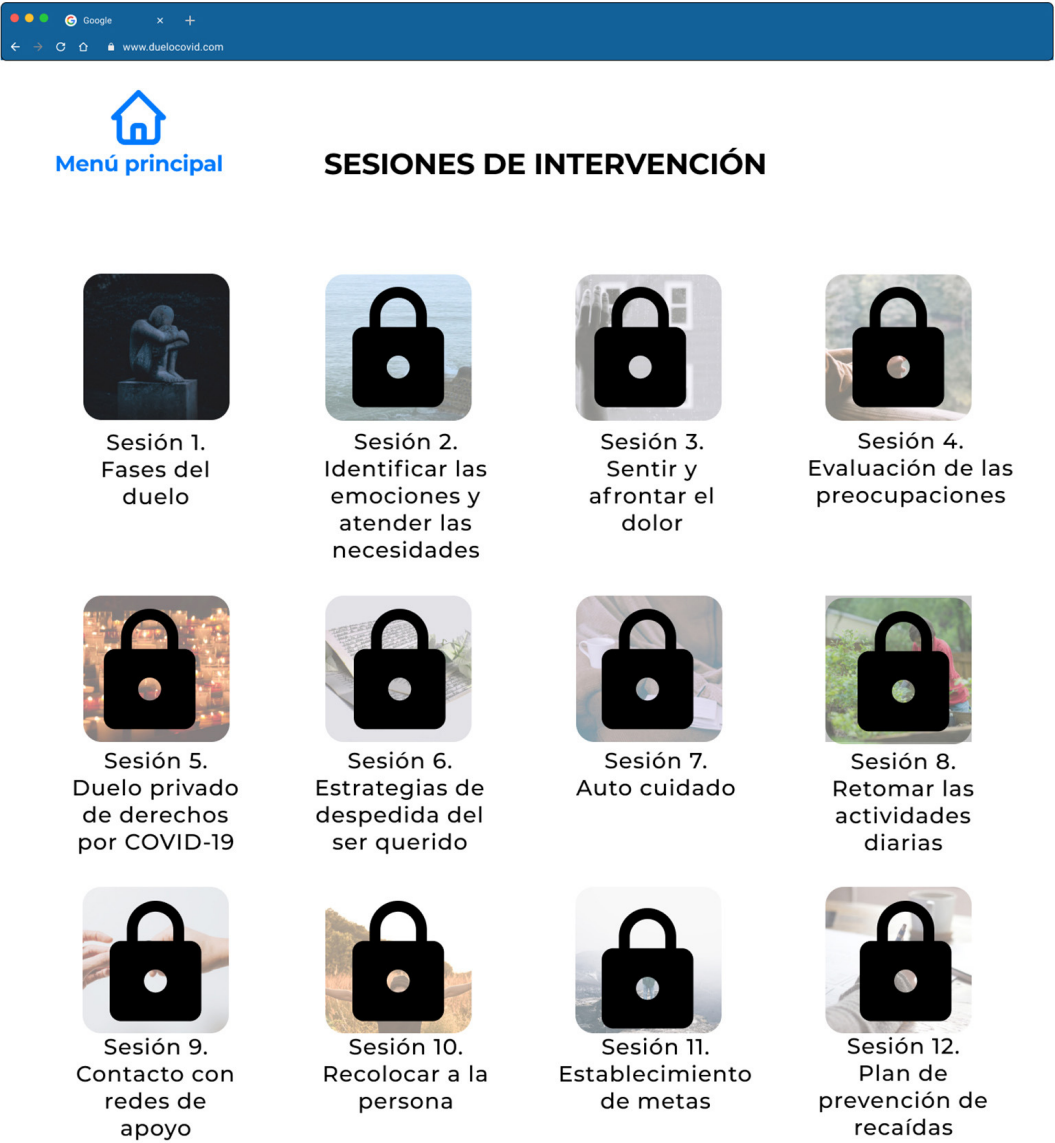
High Fidelity Prototype in Figma of the available and pending sessions page of the “Grief COVID” (Duelo COVID) intervention.

The topics of the sessions are presented to the participants from the onboarding process, and then they are explained. To go from one session to the next the participant needs to see the contents, then wait from one session to the next for at least 3 days in order to process the received contents, do the requested tasks, and answer a quiz of five multiple-answer questions (see Figure 6). Once this is done the system will activate the following session.

The process for the design of the contents of the platform was the following: a group of expert clinicians designed the sessions and then they were provided to the coordinator of these projects. After the evaluation, and correcting when necessary, these sessions were provided to a narrator that audio recorded the sessions, and to a team of seven designers that created the draft, illustrations, and animation of the videos, editing the audios and introducing them into the videos.

The platform will be delivered considering the principles of responsive design, in order to see the intervention in any device, such as a desktop, or a mobile device (Nebeling and Norrie, 2013).

In order to increase the adherence to the intervention the platform will send email reminders, a tool that has shown to be effective for this purpose (Horsch et al., 2017). Therefore, an email will be sent to the participant to notify them when the new session is open, and if the participant is absent from the platform for more than 4 days.

### Description of the Intervention

The current protocol article describes the proposal and development of a UX-based self-administered online intervention for the prevention of complicated grief disorder with Mexican population who have lost someone due to or during the COVID-19 pandemic. This intervention will consist of 12 sessions; the purpose is to identify and resolve conflicts that contribute to the risk of developing CGD, like anxiety disorders, which are an exclusion criterion, and to improve well-being in different areas of their life (physical, emotional, cognitive and spiritual). The intervention is based on CBT, BAT, Mindfulness, and PP. Through this intervention, is expected that the quality of life and perception of life satisfaction will get improve. As well, it will be looked at as a reduction of anxiety and depression symptoms and the increase of sleep quality.

Through 12 sessions, it is expected to guide the participant to continue their own process of natural adaptation to the loss with their own coping strategies, by obtaining greater knowledge about the expected manifestations, while preventing the appearance of symptoms that lead to the complication of the grief process.

Session 1, psychoeducation, will be carried out regarding the manifestations of normal grief and its phases, with the objective that participants are able to work on the emotions experienced from the loss and that they begin to adapt to the situation.

Sessions 2–4 will mainly focus on the search for emotional relief, that is, helping the person to manage the pain of loss, recognizing, and accepting the pain experienced, therefore, it is expected that they learn to elaborate and regulate their emotions to feel them, and finally, to face the duel. The person will develop the ability to name their emotions, which will allow them to stop perceiving them as something threatening, likewise, they will enhance personal resources, as well as strengths and virtues. Finally, the participants will receive resources to detect those current situations that are being difficult to handle, and they will learn how to handle the emergence of emotions and thoughts that are unpleasant in the face of such events.

Sessions 5 and 6 are focused on how losses are experienced during the COVID-19 outbreak, it will help to identify the characteristics of deaths deprived of rights, what is the emotional impact that losses have during the pandemic, and it will allow survivors to find a way to say goodbye to their loved one when they were not able to do so. In addition, it will allow the experience of pain to normalize, by recognizing thoughts that accompany their emotions, generating alternative thoughts that reduce emotional discomfort and make it more tolerable.

Sessions 7–9 are aimed at acceptance and adaptation to loss, through these, the establishment of self-care measures is promoted in the different areas of people's lives, in order to influence their gradual recovery of daily activities and to reconnect with support networks. To achieve this, they will learn to prioritize their activities and carry them out depending on the degree of difficulty that these imply for the bereaved, going from the simplest to the most complex.

Finally, sessions 10–12 are designed to work on the readjustment and recovery, that is, helping the bereaved to reposition the deceased in their life without causing suffering, encouraging him or her to resume his or her life project and life goals at the person's own pace and to establish a plan relapse prevention based on the knowledge acquired through the sessions. On Table 2, it is located the description of the main objective and the theoretical model of each of the sessions that make up the platform.

**Table 2:** Detailed description of the main objective and the theoretical model of each of the sessions of the COVID grief self-applied intervention.

**Module**	**Theory**	**Principal objective**
Grief phases	Cognitive behavioral therapy	To carry out psychoeducation about the grief process and manifestations (Neimeyer, 2014). To explain myths about grief and grief phases (Klüber-Ross, 1969; Klüber-Ross and David, 2005).
To identify emotions and attend needs	Cognitive behavioral therapy and mindfulness	To accept negative impacts and search for emotional consequences to approach them. To identify needs, difficulties, preoccupations, and emotions (Neimeyer, 2014).
To feel and face the pain	Cognitive behavioral therapy and mindfulness and positive psychology	To normalize positive emotions, expressions, and to exculpate for experimenting them (Neimeyer, 2014).
Evaluation of pre-occupations	Cognitive behavioral therapy and mindfulness	To explore resources and possibilities of coping with difficulties. To recognize signs of each emotion (Neimeyer, 2014).
Rights deprived grief for COVID-19	Cognitive behavioral therapy	To orientate on how to identify characteristics of rights deprived deaths. To evaluate the emotional impact of deaths during the pandemic (Worden, 2008; Payás, 2010; Kokou-Kpolou et al., 2020).
Parting strategies	Cognitive behavioral therapy	To guide in alternative parting rituals application when it is not possible to say goodbye. To allow for emotional expression during parting rituals (World Health Organization, 2016; Osiris et al., 2020).
Self-care	Cognitive behavioral therapy and behavioral activation	To promote actions of self-care in the different spheres of life (physical, emotional, cognitive, and spiritual) (Díaz et al., 2014; Neimeyer, 2014).
Take back daily activities	Behavioral activation	To help a person gradually come back to his/her daily activities from the simpler to the increasingly difficult ones (Díaz et al., 2014; Osiris et al., 2020). To stimulate facing the new reality, resulting from loss, and to promote developing the necessary practice tasks (Barreto and Soler, 2007).
Contact with a support network	Cognitive behavioral therapy	To highlight the importance of having a social support network to express the emotional, as well as the consequences of avoiding isolation, and recognizing the importance of having lonely moments (World Health Organization, 2016; Osiris et al., 2020).
Relocate to the deceased person	Cognitive behavioral therapy	To guide the person to continue with their life without an unbearable pain related to memories of their loved one.
Establishment of goals	Behavioral activation	To take back to short and medium lapse, which get adjusted to personal needs, considering the scope and possible obstacles (World Health Organization, 2016; Osiris et al., 2020).
Relapse prevention plan	Cognitive behavioral therapy and behavioral activation	To elaborate a personalized relapse prevention plan.

This intervention contains communication skills tools to help externalize the problem and set realistic goals (Malkinson, 2010). Among them, guiding and reassuring the person to reduce anxiety symptoms and explore emotions (Aoun et al., 2020; Morris et al., 2020); transmitting therapeutic support to process information and recognize emotion (Neimeyer, 2014); paying attention to self-care; maintenance of social relationships through the internet, mobile, etc.; help to restructure thinking; expressing and identifying emotions; recognizing positive emotions (Lyubomirsky, 2008).

Each of the 12 sessions is composed by the following structure: first, a mindfulness exercise is presented so that users are located in the present moment and participate with full awareness of the session and the experiential activities; Subsequently, a series of short psychoeducational videos are shown, of ~5 min, through which an explanation of the topics addressed in each of the sessions is given (see Table 2). In turn, it contains 2–3 experiential activities that participants are proposed to carry out based on what has been explained, using relaxation techniques, visualization, mindfulness, and/or orientation, as well as strategies to learn to name and recognize emotions, solution of problems, setting short, medium, and long-term goals, self-care, scheduling activities, activating a support network, tolerance to discomfort and prevention of relapses, depending on the content of each session. It is advisable to take 3 days between each session so that the participant can process what was addressed in each one. At the end of the session again, a mindfulness exercise is presented to the participant to close what was worked on during it. Similarly, the information contained in the sessions is available in written format for those who prefer to read the content instead of watching the videos.

### Possible Negative Effects and Strategies to Reduce the Risk or Damage for the Participants

The main objective of this study is to aid the persons that lost someone due to COVID-19 or during the pandemic, therefore producing a benefit, and not a harm. However, it has been recorded that in previous studies, negative effects had been found on internet-based interventions. For example, on the study of Boettcher et al. (2014), it was identified that from 133 participants receiving a 11-week guided treatment for Social Anxiety Treatment, that out of 19 participants, in detail from the total of participants, 5% reported the emergence of new symptoms, 4% noted a deterioration of targeted symptoms. Other side effects, less frequent, were negative well-being lack of clear treatment results, non-compliance with the treatment followed by changes in the work situation, and fear of being stigmatized. In the same line, Rozental et al. (2015) in a review from four clinical trials with a total sample of 558 participants identified that 9.3% of the participants indicated a negative effect. Among the main ones are deterioration of the targeted conditions, and new symptoms.

Due to this data, and in order to protect at all times the participants of the COVID grief self-applied intervention, the implemented controls will start from the moment they answer the questionnaires, and with how the inclusion and exclusion criteria are established, where, with strict filters, the participants need to fulfill 5 inclusion criteria points and not fulfill none of the six points of the exclusion criteria. With this, ensuring that the participants with a more serious condition would not have access to the self-applied intervention and would be directed to specialized phone numbers and email addresses where they could contact a therapist of one of the institutions in México that is offering free of charge psychological treatment via phone or video call.

Also, it will be added on the platform an option to indicate if the participants that identify that they would need more support, or for whom the self-applied intervention is not sufficient, a special email address will be indicated where the participants can write an email and they will be contacted and redirected to the same phone numbers that the excluded participants would have access to. In this sense all the participants will be protected at all times from the possible negative effects that could be related directly or indirectly to the intervention. The participants that use this option will be removed from the intervention and the data will not be included in the statistical analysis.

Finally, an open question will be added on the post-evaluation, where the participant can indicate if, even though he or she did not contact the special email address, but felt that at some point during the intervention the symptomatology worsened, and in which sense. This will provide qualitative data that could increase the knowledge about the possible negative effects related to a self-applied online interventions.

### Proposed Analyses

To test hypotheses, the Statistical Package for Social Sciences (SPSS) will be used. To examine whether a telepsychology intervention will improve quality of life and perception of the satisfaction of life, and also reduce adverse mental health indicators previously associated to grief/bereavement (e.g., depressive and anxiety symptoms), multiple (4) mixed between-within subjects ANOVA tests (Howell, 1999) will be conducted (within group comparisons; Time 1 [T1]—Pre-test, Time 2 [T2]—Post-intervention, Time 3 [T3]−3-month follow-up, Time 4 [T4]−6-month follow-up) with planned *post-hoc* tests (Tukey HSD, Gravetter and Wallnau, 2013) and between-group comparisons with experimental and control groups carried out from Time 1 to Time 4. Only complete questionnaire submissions from T1 to T4 will be considered for the proposed statistical analyses. Incomplete questionnaire submissions will not be considered for statistical analyses due to risk of bias and power reduction associated with multiple imputation methods (Field, 2009).

One-tailed analysis in this experiment means the strength of the effect is expected to be higher between T1 and T2 than between T1 and T4. That is, we expect a stronger effect during the intervention compared to the post-intervention follow-up phase. It also means the experimental group is expected to outperform the control group in terms of experiencing lower levels of adverse mental health related to bereavement, and higher levels of well-being from T2 to T4.

### Power Size Calculation

A total of 49 participants will be recruited online for this study. The number of participants and expected power size needed is based on previous internet-based interventions focused on grief and associated adverse mental health (e.g., Kersting et al., 2013), and an a priori power analysis using G^*^Power software (Mixed between-within groups ANOVA tests, 1 – β = 0.95, α = 0.05, Cohen's *d* = 0.8) (Cohen, 1973), which revealed that the study would require a total sample size of *N* = 36. According to Cohen (1988), a value of 0.80 is considered a large effect size. An additional 13 participants have been added to this estimated sample size to account for an assumed 36% dropout rate, considering Linardon and Fuller-Tyszkiewicz's (2019) mean meta-analytic attrition rate found at longer-term follow up assessments in randomized controlled trials for smartphone-delivered interventions.

## Discussion

Due to the COVID-19 pandemic, a large number of deaths have occurred throughout the world and in Mexico, which has generated multiple consequences at a psychological level related to the suffering caused by the mandatory physical separation of relatives infected with COVID-19 (Singer et al., 2020). Among the main side effects of the confinement are anxiety, post-traumatic stress, depression, suicidal behavior, addictions, and domestic violence (Mengin et al., 2020). In addition to this, due to the constant losses that people face, such as not being able to say goodbye to their loved ones or being limited to performing traditional social and cultural rituals (Goveas and Shear, 2020; Morris et al., 2020), this can lead to a complicated grief, characterized by intense emotional discomfort, which in turn could generate a disability in people's daily functioning, compromising their health with an undefined duration (Barreto et al., 2012). In this way, the need arises to give timely attention to the psychological consequences due to the COVID-19 pandemic, however, we are facing a reduction in the availability to carry out these interventions in person, so it has been opted to provide psychological support at a distance through different communication platforms (Eisma et al., 2020), that help to mitigate the impact of the COVID-19 pandemic on the mental health of the population (Blake et al., 2020).

In order to respond to the needs of the Mexican population, the objective of this study is to prevent complicated grief through a self-applied online intervention to influence the early stages of the grief process (Litz et al., 2014). Related to this, Zhang et al. (2006), proposed that clinical interventions should focus on differentiating between expected grief reactions and complicated grief reactions, detecting the risk factors that make people more vulnerable to develop grief complications and establish measures to anticipate loss-maladjustment behavior. For this reason, this work proposes the design, development and validation of a self-applied intervention based on Cognitive Behavioral Therapy, Behavioral Activation Therapy, Positive Psychology and Mindfulness.

The online modality is chosen based on the advantages that some authors have found, such as greater flexibility and anonymity compared to face-to-face therapy (Wagner et al., 2014; Hoffmann et al., 2018). Another advantage of the implementation of online interventions has to do with reaching the vulnerable and low-income population, who find it difficult to have access to a psychotherapeutic service, which is why these types of interventions are profitable and accessible (Barak and Grohol, 2011). Another self-applied intervention has been designed and implemented by the authors of this manuscript for the Mexican population in order to reduce the symptoms of anxiety and depression, and increase positive emotions and sleep quality, during and after the COVID-19 outbreak (Dominguez-Rodriguez et al., 2020).

Another advantage of the proposed intervention is the novelty of designing it following the principles of UX design, similar to the industry where tools, such as Figma (2020) and Usability Hub (2020) are applied. The UX methodology has the potential to increase the adherence of the users of the interventions, due to its design in terms of how they would want to receive this intervention. In spite of the relevance of the UX steps in order to improve online interventions, this has not been widely reported. However, few exceptions, such as the study of Wozney et al. (2015), where five clinicians and four adolescents aged <20 years old evaluated the platform before identifying learnability, technical errors and efficiency, user satisfaction, site aesthetics, among other contents, but stating the importance of evaluating the UX of the participants objective in order to determine if their program was easy to understand, efficient with relevant content, and satisfactory. However, we went a step ahead, and we evaluated the proposal of the idea of the Covid Grief platform before it existed, from when it was an idea until it became a prototype of the product. With this we reduced greatly the costs of modifying a platform with the input of the users. In low and middle-income countries where usual care for mental health problems is scarce (Fu et al., 2020), the UX approach is cost wise. Also, along with the study of Wozney et al. (2015), considering the UX can help to create a positive experience toward using the platform, therefore increasing the probability of meeting the objectives in our study, and decreasing the probability of developing Complicated Grief Disorder.

Regarding the limitations of this study, the first limitation is that a wait list control group is proposed, instead of another intervention, or instead of providing the treatment directly to all the participants that meet the inclusion criteria and that do not have any points of the exclusion criteria. With respect to these points, other options, such as face to face treatment, would not be recommended due to the safety measures needed to avoid more infections of COVID-19. Neither therapist assisted intervention would be possible due to the broad amount of participants that we would like to offer an online open intervention without any cost to all the Mexican population. Due to this, and since this intervention is an exploratory study, due to the fact that we do not have knowledge that there is another freely available, completely self-applied intervention, based on UX principles, for the prevention of complicated grief disorder, before or during the COVID-19 pandemic. Therefore, we have set a control group comparison of this randomized controlled trial, that 36 days after completing the initial evaluation, will receive the intervention and also preventive measures considered to prevent damage to the participants and previously provide explanations. Finally, regarding this limitation, articles with well-designed studies were also considered, as well as recent articles published in prestigious journals that included a wait list control group for Online or presential Psychological Interventions (Eckert et al., 2018; Hjemdal et al., 2019; Stächele et al., 2020).

The following limitation is related to the self-report instruments, where it would be of higher reliability when the measures are applied by a trained clinician. However, due to the reach of this self-applied intervention, this would not be possible with the current resources and with not having a sufficient number of therapists to apply all the measures to the participants. Therefore, this study is supported on the benefits that it provides a self-applied intervention where the participants can answer the instruments whenever they want. Regarding this limitation, the participants will answer very similar instruments before starting the intervention and after receiving the intervention, that could affect the response on the second time they answer the instruments, as previously indicated, once the participants finish the intervention they will also be requested to fill the scales Opinion on the treatment (Botella et al., 2009), and the System Usability Scale (Brooke, 1996). However, in order to fulfill the reliability of this study it would be needed to perform the evaluation with the same instruments. A solution in future studies could be to count with a controlled number of participants and with the necessary budget to be able to have an in person or an online evaluation done by a trained psychologist.

Regarding the confounding variables that could influence the results, among the main ones is the gender. As widely studied, in general, men are more reticent to search for psychological support compared to women (Liddon et al., 2018; Seidler et al., 2018), and also when they are receiving online treatment, there is a bigger dropout of men than women, however the results are still inconclusive and further research needs to be conducted (Melville et al., 2010). Therefore, it is probable that in our study most of the participants could also be women, and that the results cannot be generalized for both genders. However, in order to reduce the probability that this could happen, we will actively work on the advertisement of the intervention for everyone, and that men could also get benefits out of this intervention, making emphasis on the anonymity that the intervention provides, since it is not necessary to provide name, telephone number, address or economic status. The participant can feel safe that his or her identity will not be identified. Also, the proper statistical analysis would be applied in case that the participants are considerably more women than men, to try to reduce the impact of this difference. Another confounding variable that could influence on the results is the educational level of the participants, where participants with a higher educational level engage more with online interventions and have lower drop-out rates compared to lower educated persons. The reasons could be related to the fact that lower educated people tend to use more written health information, invest less time online seeking health information, and it is possible that they lose interest in the intervention sooner (Reinwand et al., 2015). In order to solve this, on the section “Description of the UX process for the design of the Grief COVID Platform” it was described that the platform has been designed with the highest standards of usability testing, giving as a result a platform easy to use, and therefore making it more attractive and without a struggle platform to be used. The results of the intervention would provide information regarding UX as a methodology that could be implemented to improve adherence, and attractiveness to the interventions. Also, email reminders will be sent for the open sessions and to try to “rescue” when the participants disconnect from the intervention for more than 5 days.

With respect to the unintended effects that could appear due to this treatment, they are considered and tried to be controlled on the possible side effects and controls to reduce the risk or damage for the participants section of this manuscript. Where it is explained that the platform will have several filters to grant access to the treatment, and if any of these filters is not fulfilled the participant will be directed to a list of free of charge psychological services offered by diverse institutions in México due to the pandemic. Also, in case that the participant crosses these filters but he or she feels that the intervention is not helping him or her and is feeling worse, an emergency email will be included inside the platform to reach the researchers in this project, and also the same list of institutions will be provided as excluded participants received. Once it is registered by the contact of the participant to the clinical team, the participant data will be automatically removed from the platform and will be indicated on the following reports of articles with the results of the intervention.

This intervention design is expected to improve quality of life and perception of life satisfaction, depression, anxiety, hopelessness and stress symptoms, among others, and greater sleep quality, thus coinciding with the studies by Lotzin et al. (2020) and Riva et al. (2020). The contents of Grief COVID intervention will be implemented through a responsive web application. At the time of writing this manuscript, the participants have not been evaluated nor assigned to any group (intervention or control), but the evaluation of the first proposal of the platform and the usability test have been conducted with a sample of a representative of the participants. If this research shows evidence of effectiveness, then it could be implemented in other Latin American countries with the respective cultural adaptation.

## Ethics Statement

This study will be carried out protecting the participants integrity and information. The protocol was approved by the Research Ethics Committee of the Autonomous University of Ciudad Juárez, México. All the participants will have access to the written informed consent in accordance with the Declaration of Helsinki.

## Author Contributions

AD-R conceived the original idea, did the User Experience process, and supervised the graphic designers' team and all the project steps. AD-R, SM-L, MH, AD, PA-L, EE, CA-S, and AS designed the study and the original protocol. SM-L and MH wrote the scripts for the 12 intervention sessions. JA and AD-R developed the COVID Grief platform. AD-R, SM-L, MH, AD, PA-L, EE, CA-S, AS, RV, and FR-M wrote the paper. AC narrated and audio recorded the 12 sessions. FR-M obtained the funding for the platform. All the authors contributed to the article and approved the submitted version.

## Conflict of Interest

The authors declare that the research was conducted in the absence of any commercial or financial relationships that could be construed as a potential conflict of interest.

## Abbreviations

2019-nCoV: Novel Coronavirus
BAT: Behavioral Activation Therapy
CBT: Cognitive Behavioral Therapy
CES-D: Center for Epidemiologic Studies Depression Scale
COVID-19: Coronavirus disease 2019
DASS-21: Depression Anxiety Stress Scale
GAD-7: Generalized Anxiety Disorder 7-item
PP: Positive Psychology
WHO: World Health Organization
WHOQoL: World Health Organization Quality of Life
UX: User Experience.
